# Jaynes-Gibbs Entropic Convex Duals and Orthogonal Polynomials

**DOI:** 10.3390/e24050709

**Published:** 2022-05-16

**Authors:** Richard Le Blanc

**Affiliations:** Faculté de Médecine et des Sciences de la Santé, Université de Sherbrooke 1, 3001, 12 ème Avenue Nord, Sherbrooke, QC J1H 5N4, Canada; richard.le.blanc@usherbrooke.ca

**Keywords:** noncentral distributions, orthogonal polynomials, Bayesian inference, Jaynes’ maximal entropy principle, Gibbs prior, entropic convex dual

## Abstract

The univariate noncentral distributions can be derived by multiplying their central distributions with translation factors. When constructed in terms of translated uniform distributions on unit radius hyperspheres, these translation factors become generating functions for classical families of orthogonal polynomials. The ultraspherical noncentral *t*, normal *N*, *F*, and $\chi^{2}$ distributions are thus found to be associated with the Gegenbauer, Hermite, Jacobi, and Laguerre polynomial families, respectively, with the corresponding central distributions standing for the polynomial family-defining weights. Obtained through an unconstrained minimization of the Gibbs potential, Jaynes’ maximal entropy priors are formally expressed in terms of the empirical densities’ entropic convex duals. Expanding these duals on orthogonal polynomial bases allows for the expedient determination of the Jaynes–Gibbs priors. Invoking the moment problem and the duality principle, modelization can be reduced to the direct determination of the prior moments in parametric space in terms of the Bayes factor’s orthogonal polynomial expansion coefficients in random variable space. Genomics and geophysics examples are provided.

## 1. Introduction

We shall argue that the four noncentral univariates *t*, *F*, the normal *N*, and $\chi^{2}$ distributions $\rho (r|r_{o}),$ with *r* being the one-dimensional random space variable, and $r_{o}$ the one-dimensional noncentrality parameter of the respective distributions, can all be constructed in a modular fashion by multiplying their central counterparts $\rho (r|\;r_{o}=0)$ with a factor $T(r|r_{o})$ effecting a central distribution translation, that is,

$$\rho \left(r|r_{o}\right)=T\left(r|r_{o}\right)\;\rho \left(r|0\right).$$

In statistical parlance, the noncentral distributions are needed to estimate or modelize effect sizes [1]. With the exception of the normal distribution, these translations are non-shape-preserving. The derivation of the translation factors $T(r|r_{o})$ can be carried out in two manners, depending on whether primacy is put upon translated uniform density distributions on unit radius hyperspheres, as is done in this manuscript, or on translated normal distributions, as done classically. We shall review both derivations herein, with emphasis on the hyperspherical distributions.

We choose to place primacy upon the simple uniform density distribution on the unit radius hypersphere $S^{\nu }$, where ν stands for either the dimension of the hypersphere surface $S^{\nu }\in {ℜ}^{\nu +1}$, or, equivalently, the degrees of freedom (dof) of the specified distribution. It is known that the projection of a unit radius uniform density hyperspherical distribution on $S^{\nu }$ on any given polar axis readily provides us with the central *t* distribution [2], and that such a projection converges with the central normal distribution N(0,1) with null mean when ν tends to infinity [3] (p. 59). This observation provides us with the needed building principle used throughout this manuscript: use the uniform density distribution on the unit radius hypersphere $S^{\nu }\in {ℜ}^{\nu +1}$ to derive modular expressions for the central and noncentral *t*, *F*, *N*, and $\chi^{2}$ distributions. In order to proceed, one needs to master some very simple notions concerning the hypersphere geometry. The projection of a random unit vector x on the unit radius hypersphere $S^{\nu_{2}}$ on any given unit polar axis p naturally defines a polar angle θ through the scalar product cosθ=x·p. As such, the central *t* distribution with $\nu_{2}$ degrees of freedom can be drawn on the compact support −1<cosθ<1, on which it acquires a simple expression in trigonometric terms: it is simply proportional to $\sin^{\nu_{2}-1}\theta$ after integration of the azimuthal coordinates. See Saville and Wood [2] for a very extensive digression on the subject. On the familiar sphere $S^{2}\in {ℜ}^{3}$, the latter provides us with the well-known spherical surface element sinθdθ after the integration of the azimuthal coordinate. Similarly, the central $F^{\left(\nu_{1},\;\nu_{2}\right)}$ distribution becomes proportional to $\cos^{\nu_{1}-1}\theta \;\sin^{\nu_{2}-1}\theta$ on the compact domain 0≤cosθ<1, where θ is the angle between a random vector x on the hypersphere $S^{\nu_{1}+\nu_{2}}$ and a secant hyperplane defining the subspace $S^{\nu_{1}}$. In statistical parlance, the $S^{\nu_{1}}$ and $S^{\nu_{2}}$ subspaces refer to the between-class and within-class variance spaces in an analysis variance (ANOVA), as reflected by the *F*-statistic redefinition in trigonometric terms, found in Section 2. As it turns out, these two central uniform hyperspherical distributions are all that is needed to proceed with the derivation of all the univariate noncentral *t*, *F*, *N*, and $\chi^{2}$ distributions where, for uniformity of designation, a normal distribution N(δ,1) with non-vanishing mean δ and unit variance will be simply referred to as the noncentral normal distribution. Finally, in the theory of orthogonal polynomials, the designation ultraspherical polynomials—also known as Gegenbauer polynomials [4]—has prevailed over that of hyperspherical polynomials, and we shall abide by this nomenclature. In order to distinguish the ultraspherical *t* and *F* distributions from the ones derived classically from the normal *N* and $\chi^{2}$ distributions, we shall designate the former densities by the Greek letter υ (upsilon), as in υπερσφαίρα (ypersfaíra or hypersphere in English), and the latter densities by the Greek letter ρ.

In Bayesian inference, the determination of the Bayes prior is referred to as an inverse problem, and Jaynes’ data-constrained maximal entropy priors provide a principled solution to this inverse problem [5,6,7]. The maximal entropy is reached by minimizing the Gibbs potential, and the solution to this optimization problem requires the determination of the empirical density entropic convex dual. See Le Blanc [8] for an extensive review on the subject, with references. As derived from translated normal distributions, the classical noncentral distributions all have a submodular decomposition for their translation factor of the form

$$T\left(r|r_{o}\right)=E\left(r|r_{o}\right)\;e^{-r_{0}^{2}/2},$$

with $E(r|r_{o})$ being a generalized hypergeometric function, a decomposition which does not readily provide regrouping of terms of similar order in the noncentrality parameter $r_{o}$. Conversely, the translation factors for the noncentral *t*, the normal *N*, *F*, and $\chi^{2}$ distributions, as derived from translated uniform distributions on unit radius hyperspheres, are found to be generating functions for the Gegenbauer, Hermite, Laguerre, and Jacobi orthogonal polynomial families ${\left\{P_{n}\right\}}_{n=0}^{\infty }$, respectively, and intrinsic properties of the orthogonal polynomials [9] allow for regrouping all terms of similar order in the noncentrality parameter $r_{o},$ that is,

$$T\left(r|r_{o}\right)=\sum_{n=0}^{\infty }c_{n}P_{n}\left(r\right)r_{o}^{n},$$

with the constants $c_{n}$ provided hereafter. To the best of our knowledge, the derivation of these translation factors and their identification as orthogonal polynomial family-generating functions has not been carried out before. As a consequence, one can expand the entropic convex duals on a generally small number of low-order orthogonal polynomials, an approach which greatly curtails the computational cost of determining the duals and, thus, the Jaynes–Gibbs priors. To the best of our knowledge, the expansion and discretization of the convex duals over orthogonal polynomial bases has not been proposed before. We adopt in this manuscript the convention that the polynomial family-defining weight functions should be provided by the corresponding normalized central distributions, a convention which results in much simplified expressions for the norm of the orthogonal polynomials.

In parametric Bayesian modeling, the translation factor $T(r|r_{o})$ can be weighted by a Bayesian prior $\pi (r_{o})$ to obtain the Bayes factor

$$\mathrm{BF}\left(r\right)=\int T\left(r|r_{o}\right)\;\pi \left(r_{o}\right)\;dr_{o}$$

for the generic superposition density

$$\rho \left(r\right)=\int \rho \left(r|r_{o}\right)\;\pi \left(r_{o}\right)\;dr_{o}=\int T\left(r|r_{o}\right)\;\rho \left(r|0\right)\;\pi \left(r_{o}\right)\;dr_{o}=BF\left(r\right)\;\rho \left(r|0\right).$$

We will, in this manuscript, identify the normalized central distribution ρ(r|0) with the orthogonal polynomial family-defining weight w(r)≡ρ(r|0) [9], with, as a result, the rewriting of any generic density ρ(r) as

ρ(r)=BF(r)w(r),

that is, the Bayes factor BF(r) can stand as a substitute to the generic density ρ(r). With $p\left(r\right)=\int^{r}\rho \left(r^{\prime }|0\right)dr^{\prime }$ standing for the cumulative density function of the respective central distributions ρ(r|0), or, equivalently, the *p*-value of the null hypothesis statistical testing (NHST) procedure, we have that ρ(p)=BF(p). That is, the Bayes factor BF(p) stands for the generally nonuniform *p*-value distribution ρ(p) of the above generic superposition density (see Le Blanc [10] for details of a proof), to be contrasted with the NHST framework which only considers the central distribution with its uninformative uniform *p*-value distribution. Now, if one’s goal is only to model the density BF(p) or to compute the associated local false discovery rate fdr(p)=1/(1+BF(p)) [11], one then only needs prior moments to carry on with the modelization. Invoking the moment problem [12] and the duality principle [13], prior moments in parametric space will be shown to be readily provided by Bayes factor BF(r) orthogonal polynomial expansion coefficients in random variable space—that is,

$$\int_{R_{o}}\pi \left(r_{o}\right)\;r_{o}^{n}\;dr_{o}=\frac{1}{c_{n}{∥P_{n}∥}^{2}}\int_{R}P_{n}\left(r^{\prime }\right)\;BF\left(r^{\prime }\right)\;w\left(r^{\prime }\right)\;dr^{\prime }.$$

The paper is organized as follows. In Section 2, we review, for the sake of completeness, the derivation of the classical univariate noncentral distributions as derived from translated normal distributions. In Section 3, the univariate ultraspherical noncentral *t*, the normal *N*, *F*, and $\chi^{2}$ distributions are derived from translated uniform density distributions on unit radius hyperspheres, and are shown to be expressible as products of their central distribution times specific generating functions for the Gegenbauer, Hermite, Jacobi, and Laguerre orthogonal polynomial families, respectively. We argue, in Section 4, that the determination of the Gibbs priors in terms of empirical densities’ entropic convex duals is much simplified when these duals are expanded on a small number of low-order orthogonal polynomials. We also discuss how prior moments in parametric space are directly provided by the Bayes factor orthogonal polynomial expansion coefficients in random variable space. Section 5 and Section 6 are devoted to applications in genomics and geophysics, respectively.

## 2. The Classical Noncentral Distributions

The four central *F*, $\chi^{2}$, *t*, and normal *N* distributions can be given by

(1a)υF(ν1,ν2)(θ|0)=2Γ(ν1+ν22)Γ(ν12)Γ(ν22)cosν1−1θsinν2−1θ,0≤cosθ<1,(1b)ρχ2(ν1)(r|0)=12ν1/2Γ(ν12)r(ν1−2)/2e−r/2,0≤r<∞,(1c)ρt(ν2)(θ|0)=Γ(ν2+12)Γ(12)Γ(ν22)sinν2−1θ,−1<cosθ<1,(1d)ρN(r|0)=12πe−12r2,−∞<r<∞,

respectively, where we have redefined the conventional *t* and *F* statistics so that

(2) $$t=\sqrt{\nu_{2}}\;\frac{\cos\theta }{\sin\theta },\;-1<\cos\theta <1,$$

and

(3) $$F=\frac{\nu_{2}}{\nu_{1}}\frac{\cos^{2}\theta }{\sin^{2}\theta },\;0\leq \cos\theta <1,$$

so as to re-scale the corresponding *t* and *F* distributions on the specified finite compact domains. As discussed in the Introduction, the above central *t* and *F* distributions are projections of uniform distributions on the unit radius $S^{\nu_{2}}$ and $S^{\nu_{1}+\nu_{2}}$ hyperspheres, respectively. The reasons for such rescaling will become apparent when we link in Section 3 the ultraspherical noncentral *t* and *F* distributions with the Gegenbauer and Jacobi orthogonal polynomial families, respectively, families which both have similar finite compact support domains. It is straightforward to verify that the above central *t* and *F* distributions converge with the central normal *N* and $\chi^{2}$ distributions, respectively, in the limit $\nu_{2}\to \infty$: see the discussions leading to Equations (19) and (36) below. In geometric terms, the limit $\nu_{2}\to \infty$ allows one to restrict the study of the central *t* and *F* distributions on a restricted domain around the angle θ=π/2, around which these distributions will concentrate their respective distribution weights in concordance with the notion that a high-dimensional hypersphere concentrates its surface on a narrow equatorial band at its equator [3]. In this limit, with the respective variable substitutions $r=\sqrt{\nu_{2}-1}\cos\theta$ and $r=\left(\nu_{2}-2\right)\cos^{2}\theta$, the normal *N* and $\chi^{2}$ respective central distributions’ definition domains consequently extend to infinity as cosθ→1, as reflected by the respective distribution domains provided above.

The noncentral *t* distribution has been classically derived by considering the ratio of a random variable which distributes according to a noncentral normal distribution N(δ,1) with non-vanishing mean δ, over that of a random variable distributed according to a central $\chi_{\nu_{2}}$ distribution with $\nu_{2}$ degrees of freedom. Similarly, the noncentral *F* distribution is classically derived by considering the ratio of a noncentral $\chi^{2}$ random variable with noncentrality parameter Λ and $\nu_{1}$ degrees of freedom over that of a central $\chi^{2}$ random variable with $\nu_{2}$ degrees of freedom. See, for example, Walck et al. [14] for explicit steps for such derivations. As a result, as discussed in the Introduction, the classical distributions have a submodular decomposition for their translation factor of the form $T\left(r|r_{o}\right)=E\left(r|r_{o}\right)\;e^{-r_{0}^{2}/2}$, with $E(r|r_{o})$ being a generalized hypergeometric function [15]. Upon recalling that the *F* and $\chi^{2}$ noncentral parameter Λ stands for the square of the noncentral parameter δ for the noncentral *t* and normal distributions, we have that

(4a)ρF(ν1,ν2)(θ|Λ)=EF(ν1,ν2)(θ|Λ)e−Λ/2ρF(ν1,ν2)(θ|0),0≤cosθ<1,0≤Λ<∞,(4b)ρχ2(ν1)(r|Λ)=Eχ2(ν1)(r|Λ)e−Λ/2ρχ2(ν1)(r|0),0≤r<∞,0≤Λ<∞,(4c)ρt(ν2)(θ|δ)=Et(ν2)(θ|δ)e−δ2/2ρt(ν2)(θ|0),−1<cosθ<1,−δ<δ<∞,(4d)ρN(r|δ)=EN(r|δ)e−δ2/2ρN(r|0),−∞<r<∞,−δ<δ<∞,

with the respective factors’ $E(r|r_{o})$ McLaurin expansions — computed following the steps elaborated by Walck et al. [14]—given by

(5a)EF(ν1,ν2)(θ|Λ)=Γ(ν1/2)Γ(1/2)∑jeven≥0∞Γ((j+1)/2)Γ((j+ν1)/2)Γ((j+ν1+ν2)/2)Γ((ν1+ν2)/2)(2Λcosθ)jj!(5b)=F11(ν1+ν22;ν12;Λcos2θ2),(5c)Eχ2(ν1)(r|Λ)=Γ(ν1/2)Γ(1/2)∑jeven≥0∞Γ((j+1)/2)Γ((j+ν1)/2)(Λr)j/2j!=I(ν1−2)/2(Λr)(5d)=F10(;ν12;Λr4),(5e)Et(ν2)(θ|δ)=∑j=0∞Γ((j+1+ν2)/2)Γ((1+ν2)/2)(2δcosθ)jj!=F11(ν2+12;12;δ2cos2θ2)+2δcosθΓ(ν2+22)Γ(ν2+12)F11(ν2+22;32;δ2cos2θ2),(5f)EN(r|δ)=∑j=0∞(δr)jj!=F00(;;r)=eδr(5g)=F10(;12;δ2r24)+δrF10(;32;δ2r24)=coshδr+sinhδr=eδr,

where

(6) Fqp(a1,…,ap;b1,…,bq;x)∑n=0∞(a1)n…(ap)n(b1)n…(bq)nxnn!.

stands for the generalized hypergeometric function, and where $I^{(\nu_{1}-2)/2}\left(\Lambda r\right)$ stands for the normalized modified Bessel function [16]. The respective first expansions for the multiplicative factors *E* are successively simpler versions of the Maclaurin expansion for the noncentral *F* distribution multiplicative factor $E_{F}^{\left(\nu_{1},\nu_{2}\right)}$, such that

(7) $$E_{\chi^{2}}^{\left(\nu_{1}\right)}\;\underset{\nu_{2}\to \infty }{\leftarrow }\;E_{F}^{\left(\nu_{1},\nu_{2}\right)}\;\underset{\nu_{1}=1}{\to }\;E_{t}^{\left(\nu_{2}\right)}\underset{\nu_{2}\to \infty }{\to }\;E_{N},$$

with the understanding that the respective Maclaurin summations involve either only the even integers for the *F* and $\chi^{2}$ cases, or both odd and even integers for the *t* and normal cases. It is straightforward to verify that the classical noncentral *t* and *F* distributions converge with the noncentral normal and $\chi^{2}$ distributions, respectively, in the limit $\nu_{2}\to \infty$. The expressions for the classical noncentral distributions do not readily regroup terms of similar order in their noncentrality parameter, contrarily to the ultraspherical noncentral distributions, which do so, as is discussed next.

## 3. The Ultraspherical Noncentral Distributions

Using geometrical arguments, the ultraspherical noncentral *t*-distribution for the *t*-statistic (2) on the hypersphere $S^{\nu_{2}}$ was shown by Le Blanc [10] to be given by

(8) $$\upsilon_{t}^{\left(\nu_{2}\right)}\left(\theta |\delta \right)=T_{t}^{\left(\nu_{2}\right)}\left(\theta |\delta \right)\;\upsilon_{t}^{\left(\nu_{2}\right)}\left(\theta |0\right),$$

where $\upsilon_{t}^{\left(\nu_{2}\right)}\left(\theta |0\right)$ is identical to the central *t*-distribution $\rho_{t}^{\left(\nu_{2}\right)}\left(\theta |0\right)$ given in Equation (1), where the multiplicative distribution translation factor $T_{t}^{\left(\nu_{2}\right)}\left(\theta |\delta \right)$ is given by

(9) $$T_{t}^{\left(\nu_{2}\right)}\left(\theta |\delta \right)=\frac{1-\cos\theta \cos\theta_{\delta }}{{\left[1-2\cos\theta \cos\theta_{\delta }+\cos^{2}\theta_{\delta }\right]}^{(\nu_{2}+1)/2}},$$

and where

(10) $$\cos\theta_{\delta }=\frac{\delta /\sqrt{\nu_{2}}}{\sqrt{{\left(\delta /\sqrt{\nu_{2}}\right)}^{2}+1}}=\frac{\delta }{\sqrt{\delta^{2}+\nu_{2}}},\;0<\theta_{\delta }<\pi ,$$

in terms of the noncentrality parameter δ,−∞<δ<∞. As it turns out, the translation term $T_{t}^{\left(\nu_{2}\right)}\left(\theta |\delta \right)$ is a generating function for the ultraspherical or, equivalently, Gegenbauer polynomials: redefining the variables so that x=cosθ, $z=\cos\theta_{\delta }$, and $b=(\nu_{2}-1)/2$, we have that

(11) $$\begin{array}{ccc} T_{t}^{\left(b\right)}\left(x|z\right) & = & \frac{1-xz}{{\left[1-2xz+z^{2}\right]}^{b+1}}=\sum_{n=0}^{\infty }\frac{2b+n}{2b}\;C_{n}^{\left(b\right)}\left(x\right)\;z^{n}\\ & = & {\left(1-xz\right)}^{-(2b+1)}{\;}_{2}F_{1}\left(\frac{2b+1}{2},\frac{2b+2}{2};b+\frac{1}{2};-\frac{\left(1-x^{2}\right)z^{2}}{{\left(1-xz\right)}^{2}}\right), \end{array}$$

where $C_{n}^{\left(b\right)}\left(x\right)$ are Gegenbauer polynomials with the explicit representation [4]

(12) $$C_{n}^{\left(b\right)}\left(x\right)=\sum_{k=0}^{\left\lfloor n/2\right\rfloor }\frac{{\left(-1\right)}^{k}{\left(b\right)}_{n-k}}{k!\;(n-2k)!}{\left(2x\right)}^{n-2k},$$

where the Pochhammer symbol ${\left(x\right)}_{n}$ is defined by the equality

(13) $${\left(x\right)}_{n}=\frac{\Gamma (x+n)}{\Gamma (x)}=x\left(x+1\right)\left(x+2\right)\cdots \left(x+n-1\right),$$

where $\left\lfloor y\right\rfloor$, the floor of *y*, is given by the lowest integer such that $y-1<\left\lfloor y\right\rfloor ≦y$, and where the last equality given in terms of the hypergeometric function F12 can be deduced from Rainville [17] (Equation (144.8)). The Gegenbauer polynomials are orthogonal:

(14) $$\int_{x=-1}^{1}C_{m}^{\left(b\right)}\left(x\right)\;C_{n}^{\left(b\right)}\left(x\right)\;w_{t}^{\left(b\right)}\left(x\right)\;dx=\delta_{m,n}{∥C_{n}^{\left(b\right)}∥}^{2}$$

with respect to the weight function

(15) $$w_{t}^{\left(b\right)}\left(x\right)=\frac{\Gamma (b+1)}{\Gamma \left(\frac{1}{2}\right)\Gamma \left(b+\frac{1}{2}\right)}\;{\left(1-x^{2}\right)}^{b-1/2},$$

which is identical to the central *t* distribution (1), except for the change of the variable x=cosθ. With this weight function normalization—which, note, differs from the usual weight function for the Gegenbauer polynomials [4]—the norm of the Gegenbauer polynomials simplifies to

(16) $$∥C_{n}^{\left(b\right)}{∥}^{2}=\frac{b}{b+n}\;\frac{{\left(2b\right)}_{n}}{n!},$$

with, in particular, $∥C_{0}^{\left(b\right)}{∥}^{2}=1$. Note also that Equation (11) could be used to define a generalization $T_{n}^{\left(b\right)}\left(x\right)$ for the Chebyshev polynomials of the first kind, $T_{n}^{\left(b=0\right)}\left(x\right)$, with

(17) $$\frac{1-xz}{{\left[1-2xz+z^{2}\right]}^{b+1}}=\sum_{n=0}^{\infty }T_{n}^{\left(b\right)}\left(z\right)\;z^{n}=\sum_{n=0}^{\infty }\frac{2b+n}{2b}\;C_{n}^{\left(b\right)}\left(x\right)\;z^{n},$$

which encompasses the defining equation

(18) $$\frac{1-xz}{\left[1-2xz+z^{2}\right]}=\sum_{n=0}^{\infty }T_{n}^{\left(0\right)}\left(x\right)\;z^{n}=\lim_{b\to 0}\;\sum_{n=0}^{\infty }\frac{n}{2b}\;C_{n}^{\left(\Delta \right)}\left(x\right)\;z^{n}$$

for the Chebyshev polynomials of the first kind. Setting $x=r/{\left(2b\right)}^{1/2}$ and $z=\delta /{\left(2b\right)}^{1/2}$, the central *t* distribution converges with the normal central distribution

(19) $$\upsilon_{N}\left(r|0\right)=\frac{1}{\sqrt{2\pi }}\;e^{-\frac{1}{2}r^{2}}$$

in the limit b→∞. Applying the same limit to the Gegenbauer polynomials and their generating function (11), we have that

(20) $$\begin{array}{ccc} \lim_{b\to \infty }T_{t}^{\left(b\right)}\left(x|z\right) & = & \lim_{b\to \infty }\frac{1-xz}{{\left[1-2xz+z^{2}\right]}^{b+1}}\\ & = & \lim_{b\to \infty }\sum_{n=0}^{\infty }\frac{2b+n}{2b}\;C_{n}^{\left(b\right)}\left(\frac{r}{{\left(2b\right)}^{1/2}}\right)\;{\left(\frac{\delta }{{\left(2b\right)}^{1/2}}\right)}^{n}\\ & = & \sum_{n=0}^{\infty }{\mathrm{He}}_{n}\left(r\right)\frac{\delta^{n}}{n!}=e^{r\delta -\delta^{2}/2}=T_{N}\left(r|\delta \right), \end{array}$$

where we have used the limit result [4]

(21) $$\lim_{b\to \infty }\frac{1}{{\left(2b\right)}^{n/2}}\;C_{n}^{\left(b\right)}\left(\frac{r}{{\left(2b\right)}^{1/2}}\right)=\frac{{\mathrm{He}}_{n}\left(r\right)}{n!}.$$

The multiplicative factor $T_{N}\left(r|\delta \right)$ imparts a non-vanishing mean δ to the zero mean normal distribution, since

(22) $$T_{N}\left(r|\delta \right)\;\upsilon_{N}\left(r|0\right)=e^{\delta r-\delta^{2}/2}\;\frac{1}{\sqrt{2\pi }}\;e^{-\frac{1}{2}r^{2}}=\frac{1}{\sqrt{2\pi }}\;e^{-\frac{1}{2}{\left(r-\delta \right)}^{2}}=\upsilon_{N}\left(r|\delta \right),$$

where one recognizes the generating function $e^{\delta r-\delta^{2}/2}$ for the Hermite polynomials ${\mathrm{He}}_{n}\left(r\right)$, with the explicit representation [4]

(23) $${\mathrm{He}}_{n}\left(r\right)=n!\;\sum_{\ell =0}^{\left\lfloor n/2\right\rfloor }\frac{{\left(-1\right)}^{\ell }\;r^{n-2\ell }}{2^{\ell }\ell !\;\left(n-2\ell \right)!}.$$

The latter are orthogonal with respect to their defining weight function

(24) $$w_{N}\left(r\right)=\upsilon_{N}\left(r|0\right)=\frac{1}{\sqrt{2\pi }}\;e^{-\frac{1}{2}r^{2}},$$

with norm

(25) $$\int_{r=-\infty }^{\infty }{\mathrm{He}}_{m}\left(r\right)\;{\mathrm{He}}_{n}\left(r\right)\;w_{N}\left(r\right)\;dr=\delta_{m,n}\;n!,$$

and with $∥{\mathrm{He}}_{0}{∥}^{2}=1$ in particular.

Using geometrical arguments, the ultraspherical noncentral *F*-distribution for the *F*-statistic (3) was shown by Le Blanc [10] to be given by the integral representation

(26) $$\upsilon_{F}^{\left(\nu_{1},\;\nu_{2}\right)}\left(\theta |\Lambda \right)=T_{F}^{\left(\nu_{1},\;\nu_{2}\right)}\left(\theta |\Lambda \right)\;\upsilon_{F}^{\left(\nu_{1},\;\nu_{2}\right)}\left(\theta |0\right),$$

where $\upsilon_{F}^{\left(\nu_{1},\;\nu_{2}\right)}\left(\theta |0\right)$ is identical to the central *F* distribution $\rho_{F}^{\left(\nu_{1},\;\nu_{2}\right)}\left(\theta |0\right)$ given in Equation (1), where the multiplicative distribution translation factor $T_{F}^{\left(\nu_{1},\;\nu_{2}\right)}\left(\theta |\Lambda \right)$ is given by the integral

(27) $$T_{F}^{\left(\nu_{1},\;\nu_{2}\right)}\left(\theta |\Lambda \right)=\int_{\psi =0}^{\pi }T_{F}^{\left(\nu_{1},\;\nu_{2}\right)}\left(\theta ,\psi |\Lambda \right)\;d\psi$$

with integrand

(28) $$T_{F}^{\left(\nu_{1},\;\nu_{2}\right)}\left(\theta ,\psi |\Lambda \right)=\frac{\left(1-\cos\theta \cos\psi \cos\theta_{\Lambda }\right)}{{\left[1-2\cos\theta \cos\psi \cos\theta_{\Lambda }+\cos^{2}\theta_{\Lambda }\right]}^{(\nu_{1}+\nu_{2})/2}}\;\upsilon_{t}^{\left(\nu_{1}-1\right)}\left(\psi |0\right),$$

and where

(29) $$\cos\theta_{\Lambda }=\sqrt{\Lambda /(\Lambda +\nu_{2})},\;0\leq \Lambda <\infty ,\;0\leq \cos\theta_{\Lambda }<1,$$

in terms of the noncentral parameter Λ. The special case $\nu_{1}=1$ is given by

(30) $$\upsilon_{F}^{\left(\nu_{1}=1,\;\nu_{2}\right)}\left(\theta |\Lambda \right)=\sum_{\cos\theta^{\prime }=\left[\cos\theta ,-\cos\theta \right]}\frac{1-\cos\theta^{\prime }\cos\theta_{\Lambda }}{{\left[1-2\cos\theta^{\prime }\cos\theta_{\Lambda }+\cos^{2}\theta_{\Lambda }\right]}^{(\nu_{2}+1)/2}}\;\upsilon_{t}^{\left(\nu_{2}\right)}\left(\theta |0\right).$$

Setting $a=\left(\nu_{1}-2\right)/2,\;b=\left(\nu_{2}-2\right)/2,\;x=\cos\theta ,\;\xi =\cos\psi ,\;z=\cos\theta_{\Lambda }$, and making use of the Gegenbauer polynomial-generating function expansion (11) together with the corresponding polynomials’ explicit expression (12), the integration in (27) can be carried out: we find

(31) TF(a,b)(x|z)=∫ξ=−111−xξz[1−2xξz+z2]a+b+2wt(a)(ξ)dξ=∑n=0∞a+b+1+na+b+1Fn(a,b)(x2)z2n,=∑n=0∞(−1)n(a+b+2)n(a+1)nPn(a,b)(1−2x2)z2n=(1+z2)−(a+b+2)F12(12(a+b+2),12(a+b+3);a+1;4x2z2(1+z2)2),

where the polynomials $F_{n}^{\left(a,b\right)}\left(x^{2}\right)$ which one related to the Jacobi polynomials [4], through

(32) $$F_{n}^{\left(a,b\right)}\left(x^{2}\right)={\left(-1\right)}^{n}\frac{{\left(a+b+1\right)}_{n}}{{\left(a+1\right)}_{n}}\;P_{n}^{\left(a,b\right)}\left(1-2x^{2}\right)$$

are provided with the explicit representation

(33) $$F_{n}^{\left(a,b\right)}\left(x^{2}\right)=\sum_{k=0}^{n}\frac{{\left(-1\right)}^{k}}{k!}\frac{{\left(a+b+1\right)}_{2n-k}}{{\left(a+1\right)}_{n-k}}\frac{{\left(x^{2}\right)}^{n-k}}{(n-k)!},$$

and where the last equality—deduced from Rainville [17] (Equation (132.10))—provides us with a generating function for the polynomials in terms of the hypergeometric function F12. The $F_{n}^{\left(a,b\right)}\left(x^{2}\right)$ polynomials are orthogonal with respect to the weight function

(34) $$w_{F}^{\left(a,b\right)}\left(x\right)=2\;\frac{\Gamma (a+b+1)}{\Gamma (a+1)\Gamma (b)}\;{\left(x^{2}\right)}^{a+\frac{1}{2}}\;{\left(1-x^{2}\right)}^{b},\;0\leq x\leq 1,$$

which is identical to the central *F* distribution (1), except for the change of the variable x=cosθ, with norm

(35) $$∥F_{n}^{\left(a,b\right)}{∥}^{2}=\frac{a+b+1}{a+b+1+2n}\;\frac{{\left(a+b+1\right)}_{n}{\left(b+1\right)}_{n}}{{\left(a+1\right)}_{n}\;n!},$$

and with $∥F_{0}^{\left(a,b\right)}{∥}^{2}=1$ in particular. Equation (31) can be verified to be valid for the special case (30) with a=−1/2 ($\nu_{1}=1$). In the limit $\nu_{2}\to \infty$, the central *F* distribution converges with the central $\chi^{2}$ distribution

(36) $$\upsilon_{\chi^{2}}^{\left(\nu_{1}\right)}\left(r|0\right)=\frac{1}{2^{\nu_{1}/2}\Gamma \left(\frac{\nu_{1}}{2}\right)}r^{(\nu_{1}-2)/2}e^{-r/2},\;0\leq r<\infty ,$$

which, with $a=(\nu_{1}-2)/2$, can be used as a normalized defining weight function,

(37) $$w_{\chi^{2}}^{\left(a\right)}\left(r\right)=\frac{1}{2^{a+1}}\frac{1}{\Gamma (a+1)}r^{a}e^{-r/2},\;a>-1,$$

for the Laguerre orthogonal polynomial family. The Laguerre polynomials can be given the explicit representation [4]

(38) $$L_{n}^{\left(a\right)}\left(y\right)=\sum_{\ell =0}^{n}{\left(-1\right)}^{\ell }\;\frac{{\left(a+\ell +1\right)}_{n-\ell }}{(n-\ell )!}\;\frac{y^{\ell }}{\ell !},\;0\leq y<\infty .$$

Since Equation (32) provides the noncentral *F* distribution translation factor $T_{F}^{\left(a,b\right)}\left(x|z\right)$ with an expansion in terms of the Jacobi polynomials $P_{n}^{\left(a,b\right)}\left(1-2x^{2}\right)$, and since the limit result

(39) $$\lim_{b\to \infty }P_{n}^{\left(a,b\right)}\left(1-\frac{2y}{b}\right)=L_{n}^{\left(a\right)}\left(y\right)$$

holds [4], one verifies with $x^{2}=r/2b$ and $z^{2}=\Lambda /2b$ that

(40) limb→∞TF(a,b)(x|z)=Tχ2(a)(r|Λ)=I(a)(Λr)e−Λ/2F10(;a+1;Λr4)e−Λ/2=∑n=0∞(−1)n(a+1)nLn(a)(r/2)(Λ/2)n,

which is a lesser-known expression for the noncentral $\chi^{2}$ distribution translation factor first derived by Tiku [18]. Under normalization (37) for their defining weight factor, the norm of the Laguerre polynomials $L_{n}^{\left(a\right)}\left(r/2\right)$ is given by

(41) $$\int_{r=0}^{\infty }L_{m}^{\left(a\right)}\left(r/2\right)\;L_{n}^{\left(a\right)}\left(r/2\right)\;w_{\chi^{2}}^{\left(a\right)}\left(r\right)\;dr=\delta_{m,n}\;{∥L_{n}^{\left(a\right)}∥}^{2}\;\delta_{m,n}=\frac{{\left(a+1\right)}_{n}}{n!}$$

with, in particular, $∥L_{0}^{\left(a\right)}{∥}^{2}=1.$

To summarize, the four ultraspherical noncentral *F*, $\chi^{2}$, *t*, and the normal *N* distributions are given by the product of translation factors *T*, which take the form of generating functions for specific orthogonal polynomial families, times the corresponding normalized central distributions which also stand for the corresponding polynomial family-defining weight functions. Thus, we have that

(42a)υF(a,b)(x|z)=TF(a,b)(x|z)wF(a,b)(x),0≤x<1,0≤z<1,(42b)υχ2(a)(r|Λ)=Tχ2(a)(r|Λ)wχ2(a)(r),0≤r<∞,0≤Λ<∞,(42c)υt(b)(x|z)=Tt(b)(x|z)wt(b)(x),−1<x<1,−1<z<1,(42d)υN(r|δ)=TN(r|δ)wN(r),−∞<r<∞,−∞<δ<∞,

with the normalized central distributions

(43a)wF(a,b)(x)=2Γ(a+b+1)Γ(a+1)Γ(b)(x2)a+12(1−x2)b,(43b)wχ2(a)(r)=12a+11Γ(a+1)rae−r/2,(43c)wt(b)(x)=Γ(b+1)Γ(12)Γ(b+12)(1−x2)b−1/2(43d)wN(r)=υN(r|0)=12πe−12r2,

providing the polynomial family-defining weights, and with the corresponding generating functions and orthogonal polynomial expansions given by

(44a)TF(a,b)(x|z)=(1+z2)−(a+b+2)F12(12(a+b+2),12(a+b+3);a+1;4x2z2(1+z2)2)=∑n=0∞(−1)n(a+b+2)n(a+1)nPn(a,b)(1−2x2)z2n=∑n=0∞a+b+1+na+b+1Fn(a,b)(x2)z2n,(44b)Tχ2(a)(r|Λ)F10(;a+1;Λr4)e−Λ/2=I(a)(Λr)e−Λ/2=∑n=0∞(−1)n(a+1)nLn(a)(r/2)(Λ/2)n,(44c)Tt(b)(x|z)=(1−xz)−(2b+1)F12(2b+12,2b+22;b+12;−(1−x2)z2(1−xz)2)=1−xz[1−2xz+z2]b+1=∑n=0∞2b+n2bCn(b)(x)zn,(44d)TN(r|δ)F10(;;δr)e−δ2/2=erδ−δ2/2=∑n=0∞Hen(r)δnn!.

**Figure 1:**
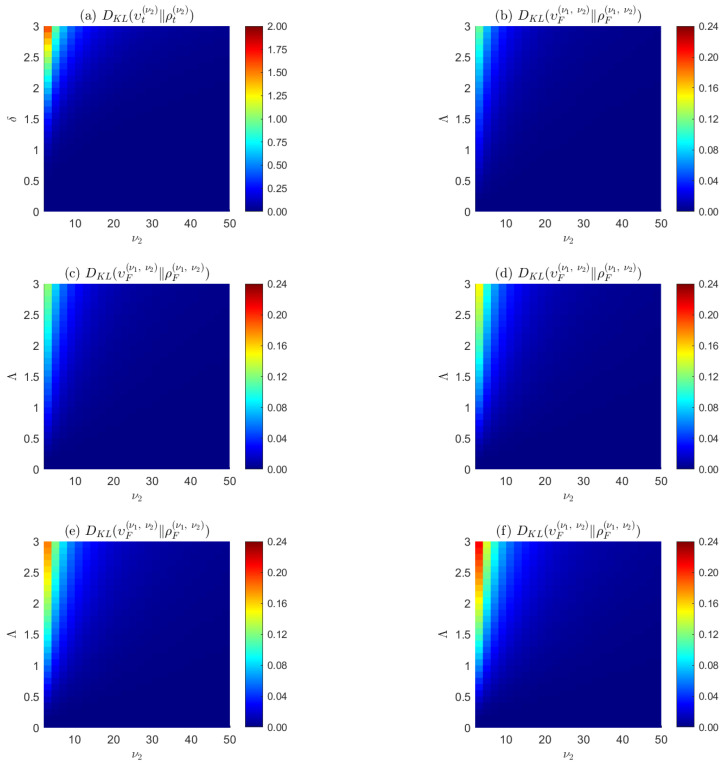
Symmetrized Kullback–Leibler divergence (**a**) between the hyperspherical $\upsilon_{t}^{\left(\nu_{2}\right)}\left(\theta |\delta \right)$ and the classical $\rho_{t}^{\left(\nu_{2}\right)}\left(\theta |\delta \right)$ noncentral *t*—distributions in the upper-left-corner plot, and (**b**–**f**) between the hyperspherical $\upsilon_{F}^{\left(\nu_{1},\nu_{2}\right)}\left(\theta |\Lambda \right)$ and the classical $\rho_{F}^{\left(\nu_{1},\nu_{2}\right)}\left(\theta |\Lambda \right)$ noncentral *F* distributions for $\nu_{1}=\left(2,\ldots ,6\right)$, respectively, for all the other plots. The ultraspherical and classical noncentral *t* and *F* distributions correspond to projections of translated uniform distributions on unit radius hyperspheres and translated normal distributions, respectively; are identical in their central distributions when their noncentrality parameters δ or Λ vanish; converge in high-dimensional (large degree-of-freedom $\nu_{2}$) spaces, but diverge in low-dimension spaces and for large noncentrality δ or Λ parameters. The ultraspherical noncentral distributions can be used as surrogates for the classical noncentral *t* and *F* distributions in high-dimensional spaces. See the text for details.

We conclude this section by stressing the fact that the random variable space for the ultraspherical noncentral distributions are translated hyperspheres rather than translated normal distributions, as is assumed for the classical noncentral distributions. The tide model developed in Section 6 provides a concrete example of such a distribution on a translated sphere. As more extensively argued in [15], the ultraspherical and classical noncentral *F* and *t* distributions correspond to projections of translated hyperspheres and translated normal distributions, respectively; are identical in their central distributions when their noncentrality parameters are zero; and converge in high-dimensional spaces, but diverge in low-dimension spaces and for large noncentrality parameters. See Figure 1. These properties ultimately stem from the counterintuitive properties of the solid hypersphere which concentrates its volume on a thin ultraspherical shell in high-dimensional spaces [3], allowing one to use the ultraspherical distributions as surrogates for the classical noncentral *t* and *F* distributions in high-dimensional spaces.

## 4. Entropic Convex Duals Expansion in Terms of Orthogonal Polynomials

Bayes–Jaynes–Gibbs data-constrained maximal entropy priors—simply designated as Gibbs priors in the following—can be objectively computed for dense datasets. See Le Blanc [8] for an extensive review on the subject, from which we recall that the Bayes–Laplace prior and posterior update rules are rooted in the convex geometry of Shannon’s entropy function, with the Kullback–Leibler relative entropy being a Bregman divergence defined in terms of the former. The Gibbs priors can be formally expressed as

(45) $$\pi \left(r_{o}\right)=\frac{1}{Z(\lambda )}\exp\left(\int_{R}\lambda \left(r\right)\upsilon \left(r|r_{o}\right)\;dr\right),$$

where the partition function Z(λ) is given by

(46) $$Z\left(\lambda \right)=\int_{R_{o}}\exp\left(\int_{R}\lambda \left(r\right)\upsilon \left(r|r_{o}\right)\;dr\right)dr_{o},$$

and where λ(r) is the entropic convex dual of the empirical density υ(r). The latter is obtained through the unconstrained minimization of the Gibbs potential

(47) $$\underset{\lambda }{\inf}G_{\rho }\left(\lambda \right)=\underset{\lambda }{\inf}\left[\log Z\left(\lambda \right)-\int_{R}\lambda \left(r\right)\upsilon \left(r\right)\;dr\right],$$

which, by convex duality, corresponds to Jaynes data-constrained maximal entropy. The corresponding Gibbs–Jaynes model for a generic empirical density υ(r) is given by

(48) $$\upsilon \left(r\right)=\int_{R_{o}}\upsilon \left(r|r_{o}\right)\;\pi \left(r_{o}\right)\;dr_{o}=\upsilon \left(r|0\right)\;\int_{R_{o}}T\left(r|r_{o}\right)\;\pi \left(r_{o}\right)\;dr_{o}=\upsilon \left(r|0\right)\;BF\left(r\right).$$

As posed, solving for the entropic convex dual function λ(r) in (47) requires one to compute its value across the entire support domain of the empirical distribution υ(r), a task which can be expensive in computing terms. Now, since the ultraspherical noncentral distribution translation factors $T(r|r_{o})$ are generating functions for orthogonal polynomial families, and since the corresponding central distributions υ(r|0) are the family-defining weight functions w(r)≡υ(r|0), one can rewrite the unconstrained minimization problem in simpler terms. Indeed, the exponentiated term in the partition function (46) can be rewritten as

(49) $$\begin{array}{ccc} \int_{R}\lambda \left(r\right)\;\upsilon \left(r|r_{o}\right)\;dr & = & \int_{R}\lambda \left(r\right)\;T\left(r|r_{o}\right)\;w\left(r\right)\;dr=\int_{R}\lambda \left(r\right)\left[\sum_{n=0}^{\infty }c_{n}P_{n}\left(r\right)\;r_{o}^{n}\right]w\left(r\right)\;dr\\ & = & \sum_{n=0}^{\infty }c_{n}\left[\int_{R}\lambda \left(r\right)\;P_{n}\left(r\right)\;w\left(r\right)\;dr\right]\;r_{o}^{n}=\sum_{n=0}^{\infty }c_{n}\lambda_{n}{∥P_{n}∥}^{2}\;r_{o}^{n}\\ & = & \sum_{n=0}^{\infty }{\tilde{\lambda }}_{n}\;r_{o}^{n}\;\mathrm{with}\;{\tilde{\lambda }}_{n}=c_{n}\lambda_{n}{∥P_{n}∥}^{2}, \end{array}$$

where the polynomials $P_{n}$, with their respective multiplicative coefficients $c_{n}$, are listed in (44), and where the $\lambda_{n}$ are the entropic convex dual expansion coefficients on family-wise orthogonal polynomials that remain to be determined. Similarly, the additive constraint term in (47) can be rewritten as

(50) $$\int_{R}\lambda \left(r\right)\;\upsilon \left(r\right)\;dr=\sum_{n=0}^{\infty }\;\lambda_{n}\int_{R}\;P_{n}\left(r\right)\;\upsilon \left(r\right)\;dr=\sum_{n=0}^{\infty }\;\lambda_{n}\int_{R}\;P_{n}\left(r\right)\;BF\left(r\right)\;w\left(r\right)\;dr=\sum_{n=0}^{\infty }\;{\tilde{\lambda }}_{n}{\tilde{\beta }}_{n},$$

where υ(r) is the empirical density to be modeled, and where

(51) $${\tilde{\beta }}_{n}=\frac{1}{c_{n}{∥P_{n}∥}^{2}}\int_{R}\;P_{n}\left(r\right)\;BF\left(r\right)\;w\left(r\right)\;dr,$$

with, in particular, ${\tilde{\beta }}_{0}=1$. Within the division by the factor $c_{n}$, the latter equality is the Bayes factor BF(r) expansion on the orthogonal polynomials in random sample space. The unconstrained optimization problem can thus be reformulated as

(52) $$\underset{\left\{{\tilde{\lambda }}_{n}\right\}}{\inf}\left[\log\left(\int_{R_{o}}\exp\left(\sum_{n=0}^{\infty }{\tilde{\lambda }}_{n}r_{o}^{n}\right)\;dr_{o}\right)-\sum_{n=0}^{\infty }{\tilde{\lambda }}_{n}{\tilde{\beta }}_{n}\right]$$

in terms of orthogonal polynomial expansion coefficient sets ${\left\{{\tilde{\lambda }}_{n}\right\}}_{n=0}^{\infty }$ for the continuous entropic convex dual function λ(r), sets which can be restricted to a small finite number of coefficients, as can be assessed by the Kullback–Leibler divergence between the empirical density and its model in terms of orthogonal polynomials. At the minimum of the Gibbs potential (52), one has the simple condition

(53) $$\frac{\int_{R_{o}}\exp\left(\sum_{n}{\tilde{\lambda }}_{n}r_{o}^{n}\right)\;r_{o}^{n}\;dr_{o}}{\int_{R_{o}}\exp\left(\sum_{n}{\tilde{\lambda }}_{n}r_{o}^{n}\right)\;dr_{o}}=\int_{R_{o}}\pi \left(r_{o}\right)\;r_{o}^{n}\;dr_{o}={\tilde{\beta }}_{n},$$

which states that the $n^{\mathrm{th}}$ moment of the noncentrality parameter $r_{o}$, as weighted by the Gibbs prior

(54) $$\pi \left(r_{o}\right)=\frac{\exp(\sum_{n}{\tilde{\lambda }}_{n}r_{o}^{n})}{\int_{R_{o}}\exp\left(\sum_{n}{\tilde{\lambda }}_{n}r_{o}^{n}\right)\;dr_{o}},$$

is, within the factor $c_{n}$, equal to the $n^{\mathrm{th}}$ coefficient of the Bayes factor expansion (51) on the orthogonal polynomial basis in random sample space. In practical terms, the determination of the Gibbs prior in terms of a small number of polynomial expansion coefficients ${\tilde{\lambda }}_{n}$ for the entropic dual convex results in a substantial reduction in the computing time needed to find the Gibbs potential minimum.

Once obtained, the Gibbs prior can be used to modelize the empirical density υ(r) according to Equation (48), in which the central distribution υ(r|0) stands for the weight function w(r) for the ultraspherical distributions listed in (42). A generic density υ(r) can thus be expanded as

(55) $$\begin{array}{ccc} \upsilon (r) & = & \int_{R_{o}}\;\upsilon \left(r|r_{o}\right)\;\pi \left(r_{o}\right)\;dr_{o}=w\left(r\right)\;\int_{R_{o}}T\left(r|r_{o}\right)\;\pi \left(r_{o}\right)\;dr_{o}\\ & = & w\left(r\right)\;\int_{R_{o}}\left[\sum_{n=0}^{\infty }c_{n}P_{n}\left(r\right)\;r_{o}^{n}\right]\;\pi \left(r_{o}\right)\;dr_{o}=w\left(r\right)\;\sum_{n=0}^{\infty }c_{n}\left[\int_{R_{o}}r_{o}^{n}\;\pi \left(r_{o}\right)\;dr_{o}\right]\;P_{n}\left(r\right)\\ & = & w\left(r\right)\;\sum_{n=0}^{\infty }c_{n}{\tilde{\beta }}_{n}P_{n}\left(r\right)=w\left(r\right)\;\sum_{n=0}^{\infty }\left[\frac{1}{∥P_{n}{∥}^{2}}\int_{R}\;P_{n}\left(r^{\prime }\right)\;BF\left(r^{\prime }\right)\;w\left(r^{\prime }\right)\;dr^{\prime }\right]\;P_{n}\left(r\right) \end{array}$$

where we have invoked the optimality condition (53) in going from the second to the third line. This sequence of equalities provides us with two alternative ways,

(56a)BF(r)=υ(r)w(r)=∫RoT(r|ro)π(ro)dro(56b)=∑n=0∞cn∫Roronπ(ro)droPn(r)=∑n=0∞1∥Pn∥2∫RPn(r′)BF(r′)w(r′)dr′Pn(r),

to model the Bayes factors and the empirical densities: either one determines the Gibbs prior $\pi (r_{o})$ via the optimization problem (52) and uses it to weigh the analytical orthogonal polynomial-generating functions $T(r|r_{o})$ listed in (44); or, one directly determines the prior moments in parametric space in terms of the Bayes factor BF(r) expansion coefficients in random variable space, as per the last equality above. The latter strategy amounts to the construction of a probability distribution in terms of its moments [12]. In that respect, recall the Hausdorff moment problem, which stipulates that the collection of all moments of a probability distribution on a bounded interval uniquely determine the distribution [19], and the Hamburger moment problem, which considers the uniqueness of solutions for the same problem on the unbounded real line [20].

Numerical exploration indicates that the convergence of the polynomial expansion for the ultraspherical noncentral *t* and *F* distributions’ generating functions is affected by the Gibbs phenomenon at the extremities of their finite noncentrality parameter ranges [21]. For this reason, when considering the noncentral *t* and *F* distributions, one can either solve for the Gibbs prior to weigh the corresponding analytic generating functions (44) in constructing the models, or use the large *b* limit for the distributions which converge with the noncentral normal *N* and $\chi^{2}$ distributions, respectively, together with the associated Hermite and Laguerre orthogonal polynomial families, to directly compute the prior moments in parametric space in terms of the Bayes factor expansion coefficients in random variable space, as according to (56). Both strategies are found to be unaffected by the Gibbs effect, as exemplified in the next section.

## 5. Genomics Examples

**Figure 2:**
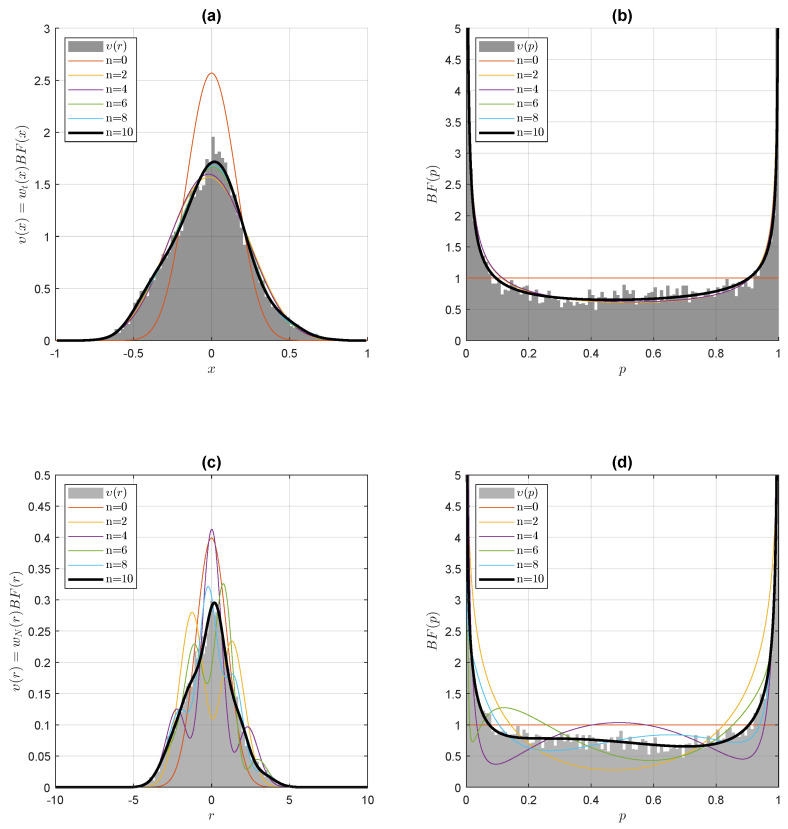
Empirical random space densities and NHST *p*-value densities modelization for the head and neck cancer dataset. The upper panels illustrate the Jaynes–Gibbs model $\upsilon \left(x\right)=w_{t}\left(x\right)\int T\left(x|z\right)\pi \left(z\right)dz$, as provided by the Gibbs prior (54) and the analytical generating function (11) for the Gegenbauer polynomials. (**a**) Upper left-hand panel: convergence of the Jaynes–Gibbs model with the empirical density $\upsilon \left(x\right)=w_{t}\left(x\right)\;BF\left(x\right)$. (**b**) Upper right-hand panel: corresponding NHST *t*-test *p*-value model densities, as modeled by the Bayes factor BF(p). As can be observed, convergence on the empirical densities is rapidly achieved with the expansion of the entropic convex dual λ(x) on a small number *n* of Gegenbauer polynomials. In the lower panels, with the Hermite polynomials standing for the Gegenbauer polynomials in the large *b* limit (21), the Bayes factor BF(r) expansion coefficients in random variable space directly provides the Gibbs prior moments $\int \pi \left(\delta \right)\delta^{n}d\delta$ in parametric space, as according to (56). (**c**) Lower left-hand panel: cumulative orthogonal polynomial expansion of the Bayes factor BF(r) in random variable space. (**d**) Lower right-hand panel: corresponding NHST central normal distribution *p*-value densities as modeled by the Bayes factor BF(p). As can be observed, convergence on the empirical densities is rapidly achieved with a small number *n* of low-order Hermite polynomials.

We first revisit, in this section, the NCBI Gene Expression Omnibus head and neck squamous cell carcinoma microarray dataset produced by [22], pertaining to 22 paired samples (N=44, $\nu_{2}=42,\;$b=41/2) of normal versus cancerous tissues, and interrogating 11,302 genes via 2-sample *t*-tests. In Le Blanc [10], a maximal entropy prior equal to the central ultraspherical *t* distribution (1) was postulated. In Le Blanc [8], the entropic dual convex λ(x) was computed on the full random variable range −1<x<1. Here, the Bayes factor is provided with two models. The first model $\upsilon \left(x\right)=w_{t}\left(x\right)\int T\left(x|z\right)\pi \left(z\right)dz$ is given in terms of the Gibbs prior π(z) (54) and the analytical Gegenbauer polynomial-generating function T(x|z) (11). We assessed the computational gains in our determination of the entropic convex dual λ(x) via Equation (52): with the empirical distribution υ(x) binned in 200 bins, and using the MATLAB^®^ optimization subroutine fminunc in a 64-bit Windows environment on a PC with an Intel(R) Core(TM) i9-9900K CPU @ 3.60 GHz processor and 64 GB of RAM, the determination times for the entropic convex dual λ(x) in terms of a small number of Gegenbauer polynomial coefficients were curtailed by a factor ranging from 100 to 1000, compared to previous determination times of λ(x) on the full random variable range −1<x<1, with elapsed times as short as a few tenths of a second. In the second model, according to (56) and with the Hermite polynomials standing for the Gegenbauer polynomials in the large *b* limit, the Bayes factor BF(r) expansion in random variable space on the Hermite polynomials provides us with the Gibbs prior moments $\int \pi \left(\delta \right)\delta^{n}d\delta$ in parametric space. As can be seen in Figure 2, the convergence of the two models with the empirical distribution is rapidly achieved with ten coefficients or less.

**Figure 3:**
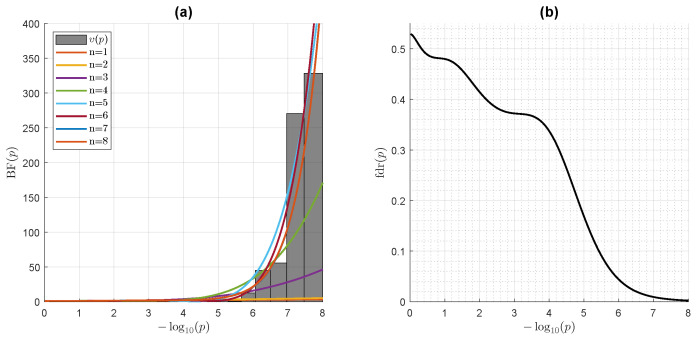
Bayes Factor BF(p) modeling a NHST *p*-value distribution from a genome-wide association study (GWAS) dataset. The GWAS compared 2244 critically ill patients with COVID-19 with 3 times as many ancestry-matched control individuals. The dataset comprises 4,380,209 $\chi_{\nu_{1}=1}^{2}$ *r*-statistics, accounting for all the SNPs in the set, which have been modelized by a logistic regression model and tested for statistical significance. (**a**) Left panel: Accrual of the successive Laguerre polynomial expansion terms in Equation (56) for the Bayes factor demonstrating an incrementally better fit of the *p*-value empirical density which strongly deviates from the NHST null hypothesis U(0,1) in the low *p*-value range. (**b**) Right panel: Local false discovery rate fdr(p)=1/(1+BF(p)). The Bayesian-based fdr crosses the 0.01 threshold (i.e., a fdr of 1%) when the NHST *p*-value reaches about $10^{-7}$ ($-\log_{10}\left(p\right)=7$), in close concordance with the threshold of significance of $5\times 10^{-8}$ ($-\log_{10}\left(p\right)=7.3$) chosen by the authors.

Next, we study a genome-wide association study (GWAS) dataset. A GWAS is an observational study assessing a genome-wide set of genetic variants in different individuals, and seeking to identify statistically significant variant-trait associations. Such studies commonly focus on associations between single-nucleotide polymorphisms (SNPs) and traits. We retrieved the GenoMICC EUR vs. UK biobank controls dataset from the GenOMICC (Genetics Of Mortality In Critical Care) GWAS, comparing 2244 critically ill patients with COVID-19 from UK intensive care units with European ancestry-matched control individuals selected from the large population-based cohort of the UK Biobank [23]. A logistic regression model was used for each of the 4,380,209 SNPs individually tested for statistical significance. We computed the empirical density of all the statistical test-associated $\chi_{\nu_{1}=1}^{2}$ *r*-statistics, and used it to compute the first eight terms of the direct polynomial expansion (56) on Laguerre polynomials for the Bayes Factor BF(p(r)). As illustrated in Figure 3, the accrual of the successive polynomial expansion terms allows for an incrementally better fit of the *p*-value empirical density, which strongly deviates from the NHST null hypothesis U(0,1) in the low *p*-value range where associations are detected. In the Bayesian framework, NHST statistical significance is replaced by the strength of Bayesian evidence, as assessed by magnitude of BF(p), which, in turn, allows for the computation of a local false discovery rate [11]

(57) fdr(p)=1/(1+BF(p))

as is also illustrated in Figure 3.

## 6. Geophysics Examples

**Figure 4:**
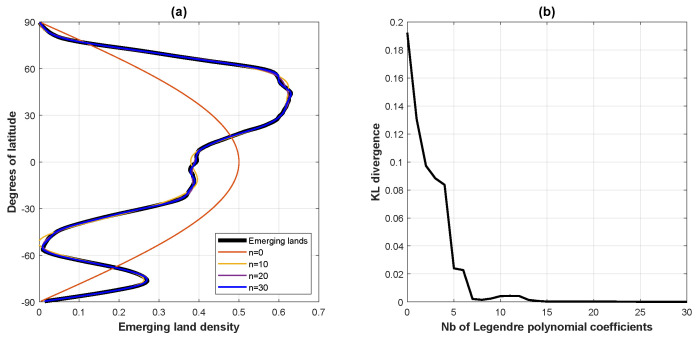
Earth’s emerging land/ice latitudinal density. Orthogonal Legendre $P_{n}$ polynomial (Gegenbaeur $C_{n}^{\left(1/2\right)}$ polynomial) modeling of Earth’s emerging land/ice masses’ latitudinal density. (**a**) Left panel: orthogonal Legendre $P_{n}$ polynomial modeling (55) of Earth’s emerging land/ice latitudinal density as expanded on the first 30 Legendre polynomials. (**b**) Right panel: Kullback–Leibler divergence between the empirical density and the model as a function of the number of orthogonal Legendre polynomials accrued.

We begin this section by modeling, in Figure 4, Earth’s above-sea-level emerging land/ice latitudinal density [24]. Because an ideal Earth surface can be described by the sphere $S^{\nu_{2}=2}$, we have that the parameter $b=(\nu_{2}-1)/2=1/2$, with the weight factor $w_{t}^{\left(1/2\right)}\left(x\right)$ in (15) reducing to the constant 1/2. As a consequence, Equation (55) is equivalent to a standard expansion on Legendre polynomials—equivalently, Gegenbauer $C_{n}^{\left(1/2\right)}$ polynomials—except for the fact that our convention regarding the central distribution normalization $w_{t}^{\left(1/2\right)}\left(x\right)=1/2$ (rather than the usual weight w(x)=1) on the span [−1,1] ensures that expansion (55) defines a normalized density.

**Figure 5:**
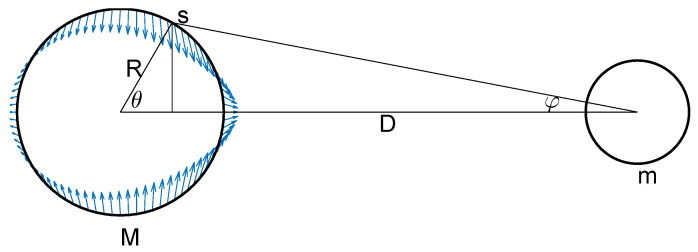
Tide geometry. Idealized model describing the gravitational tidal pull of an ideal Moon of mass *m* on a thin water layer covering an ideal Earth of mass *M* and radius *R* at distance *D* from the Moon. The horizontal tidal force is given by the modular translation factor $T_{t}^{\left(\nu 2=2\right)}\left(\theta |\delta \right)$ (9), defining on $S^{2}$ the noncentral spherical distribution $\upsilon_{t}^{\left(\nu_{2}=2\right)}\left(\theta |\delta \right)$ (8) with $\cos\theta_{\delta }=R/D$, minus a factor of one, accounting for the centrifugal force induced by the Moon–Earth system revolving around its center of gravity. The equatorial bulges are not symmetric in this purely geometrical model. The noncentrality parameter $\cos\theta_{\delta }$ is set to an unrealistic value of 0.2 to enhance the visualization of the geometrical distribution of the tidal forces.

We conclude with an example in which the translating factor (9) for the ultraspherical noncentral *t* distribution is kept as-is, instead of using its expansion (11) in terms of Gegenbauer polynomials, to argue that the noncentral spherical *t* distribution (8) on $S^{2}$ can be used to describe the geometry of the gravity forces of a simple tide model. Consider Figure 5, which describes the gravitational pull of an ideal Moon of mass *m* on the thin water layer covering an ideal Earth of mass *M* and radius *R* at distance *D* from the Moon. The gravitational tidal force T per unit mass on point *s* on the Earth’s surface is given by

(58) $$T=\frac{Gm}{{\left(D-R\cos\theta \right)}^{2}+R^{2}\sin^{2}\theta }=\frac{Gm}{D^{2}-2DR\cos\theta +R^{2}}.$$

The horizontal and vertical components of that force are given by

(59) $$\begin{array}{ccc} T_{h} & = & +\frac{Gm}{D^{2}-2DR\cos\theta +R^{2}}\times \cos\varphi =+Gm\frac{D-R\cos\theta }{{\left[D^{2}-2DR\cos\theta +R^{2}\right]}^{3/2}}, \end{array}$$

(60) $$\begin{array}{ccc} T_{v} & = & -\frac{Gm}{D^{2}-2DR\cos\theta +R^{2}}\times \sin\varphi =-Gm\frac{R\sin\theta }{{\left[D^{2}-2DR\cos\theta +R^{2}\right]}^{3/2}}, \end{array}$$

respectively. Setting our physical units so that $Gm/D^{2}=1$, and defining $\cos\theta_{\delta }=R/D$, we have that

(61) $$\begin{array}{ccc} T_{h} & = & \frac{1-\cos\theta \cos\theta_{\delta }}{{\left[1-2\cos\theta \cos\theta_{\delta }+\cos^{2}\theta_{\delta }\right]}^{3/2}}=T_{t}^{\left(\nu_{2}=2\right)}\left(\theta |\delta \right), \end{array}$$

(62) $$\begin{array}{ccc} T_{v} & = & \frac{-\sin\theta }{{\left[1-2\cos\theta \cos\theta_{\delta }+\cos^{2}\theta_{\delta }\right]}^{3/2}}. \end{array}$$

The horizontal tidal force component $T_{h}$ is thus simply given by the noncentral spherical *t* distribution translation factor $T_{t}^{\left(\nu_{2}=2\right)}\left(\theta |\delta \right)$, as provided by Equation (4). One can integrate this horizontal tidal force over the entire spherical shell by weighing it with the central spherical distribution $\upsilon_{t}^{\left(\nu_{2}=2\right)}\left(\theta |0\right)$ to account for the spherical geometry of the Earth. Since the integrand to this integral corresponds to the noncentral spherical *t* distribution $\upsilon_{t}^{\left(\nu_{2}=2\right)}\left(\theta |\delta \right)=T_{t}^{\left(\nu_{2}=2\right)}\left(\theta |\delta \right)\;\upsilon_{t}^{\left(\nu_{2}=2\right)}\left(\theta |0\right)$ on $S^{2}$, the tidal force integrates to one in our unit system. In order for the system to be stationary, an opposing force must be opposed to this integrated tidal force of one. The inertial centrifugal force originating from the joint rotation of the Moon and Earth around their center of mass plays such a role. It is evaluated to be given by $-Gm/D^{2}=-1$ in our unit system [25], which we add to the normalized horizontal tidal force $T_{h}$ above. We have plotted the resulting force field in Figure 5, with the noncentrality parameter $\cos\theta_{\delta }$ set to an unrealistic value of 0.2 to enhance the visualization of the geometrical distribution of the tidal forces. Unlike most illustrative tide diagrams with symmetrical bulges found in the literature, our tide diagram, together with its unsymmetrical equatorial water bulges, provides a more accurate visual depiction of the expected asymmetry of the tidal forces resulting from purely geometrical considerations.

## 7. Discussion

We have seen that the univariate noncentral distributions can be constructed in a modular fashion by multiplying their central distributions with specific translation factors. Using geometrical arguments, we have found that the translation factors for the ultraspherical noncentral *t*, normal, *F*, and $\chi^{2}$ distributions stand for generating functions for the Gegenbauer, Hermite, Jacobi, and Laguerre polynomial families, respectively, with their central distributions standing for the corresponding polynomial family-defining weights. These developments clearly link four of the most important classical continuous probability distributions with the powerful orthogonal polynomial formalism. To the best of our knowledge, the derivation of these translation factors and their identification as orthogonal polynomial family-generating functions has not been carried out before.

Jaynes’ maximal entropy prior is obtained through the unconstrained minimization of the Gibbs potential. In parametric Bayesian inference, the formal expression for the Gibbs potential comprises an integral of the product of the empirical distribution’s entropic convex dual λ(r) times the parametric kernel, or, equivalently, the likelihood function $\rho (r|r_{o}).$ In the case of the ultraspherical noncentral distributions, the latter integral yields discretized expansion coefficients of the entropic convex duals on orthogonal polynomial bases. The determination of the entropic convex duals is thus reduced to the much simpler and computationally economical determination of a few low-order orthogonal polynomial coefficients. By invoking the moment problem and the duality principle, prior moments in parametric space are equated with Bayes factors expansion coefficients over orthogonal polynomial bases in random variable space. To the best of our knowledge, the expansion and discretization of convex duals over orthogonal polynomial bases has not been proposed before. In an approach which bears some similarities to our work, Alibrandi and Ricciardi [26] proposed a moment-based approach, the use of a discretized kernel set ${\left\{\rho \left(r|r_{i}\right)\right\}}_{i=1}^{N}$, and the use of Jaynes’ maximal entropy principle to determine the set’s weighting factors $\left\{\pi_{i}\;|\;\sum_{i=1}^{N}\pi_{i}=1\right\}$. While their kernel discretization procedure is an ad hoc procedure, our kernel discretization is principled since it is based on the powerful orthogonal polynomial formalism.

The machine learning community has begun to exploit the classical orthogonal polynomial formalism. A non-exhaustive review identifies the use of orthogonal polynomials in optical character recognition [27], in support vector machine kernel construction [28], and in polynomial-based iteration methods for symmetric linear systems [29,30]. In a similar vein, we hope to contribute the present formalism to both the statistical and the machine learning communities.

## Data Availability

For the head and neck cancer microarray dataset, please refer to [31]. The COVID-19 GWAS dataset was accessed at https://genomicc.org/data, accessed on 3 January 2022. For Earth’s emerging land/ice latitudinal density, please refer to [32].

## Abbreviations

The following abbreviations are used in this manuscript:

ANOVA	analysis of variance
dof	degrees of freedom
FDR	false discovery rate
GWAS	genome-wide association study
NHST	null-hypothesis statistical testing
SNP	single-nucleotide polymorphism
